# ESF-YOLO: an accurate and universal object detector based on neural networks

**DOI:** 10.3389/fnins.2024.1371418

**Published:** 2024-04-08

**Authors:** Wenguang Tao, Xiaotian Wang, Tian Yan, Zhengzhuo Liu, Shizheng Wan

**Affiliations:** ^1^Unmanned System Research Institute, Northwestern Polytechnical University, Xi’an, China; ^2^Shanghai Electro-Mechanical Engineering Institute, Shanghai, China

**Keywords:** neural network, object detection, cross-scale feature fusion, attention mechanism, lightweight decoupled head, dynamic loss function

## Abstract

As an excellent single-stage object detector based on neural networks, YOLOv5 has found extensive applications in the industrial domain; however, it still exhibits certain design limitations. To address these issues, this paper proposes Efficient Scale Fusion YOLO (ESF-YOLO). Firstly, the Multi-Sampling Conv Module (MSCM) is designed, which enhances the backbone network’s learning capability for low-level features through multi-scale receptive fields and cross-scale feature fusion. Secondly, to tackle occlusion issues, a new Block-wise Channel Attention Module (BCAM) is designed, assigning greater weights to channels corresponding to critical information. Next, a lightweight Decoupled Head (LD-Head) is devised. Additionally, the loss function is redesigned to address asynchrony between labels and confidences, alleviating the imbalance between positive and negative samples during the neural network training. Finally, an adaptive scale factor for Intersection over Union (IoU) calculation is innovatively proposed, adjusting bounding box sizes adaptively to accommodate targets of different sizes in the dataset. Experimental results on the SODA10M and CBIA8K datasets demonstrate that ESF-YOLO increases Average Precision at 0.50 IoU (AP50) by 3.93 and 2.24%, Average Precision at 0.75 IoU (AP75) by 4.77 and 4.85%, and mean Average Precision (mAP) by 4 and 5.39%, respectively, validating the model’s broad applicability.

## 1 Introduction

Object detection, as one of the most crucial and challenging branches in computer vision, has been widely applied in people’s daily lives, such as in surveillance security and autonomous driving (Zaidi et al., 2022). Target detection algorithm goes through two periods, traditional target detection algorithm and deep target detection algorithm. Traditional object detection algorithms usually adopt manually designed operators to extract corresponding features, such as Deformable Part Models (DPM) (Felzenszwalb et al., 2008). However, traditional algorithms often encounter bottlenecks when dealing with complex backgrounds in images, because the artificially designed operators usually have some flaws that are hard to intuitively observe, such as the difficulty in effectively extracting abstract features. Subsequently, the development of traditional object detection algorithms stagnated, and the detection performance was hard to further improve (Hong, 2023). In recent years, object detection algorithms based on deep learning have prospered, giving rise to a series of superior methods, including but not limited to Fast R-CNN (Girshick, 2015), SSD (Liu et al., 2016), Faster R-CNN (Ren et al., 2017), RetinaNet (Lin et al., 2017), EfficientDet (Tan et al., 2020), CornerNet (Law and Deng, 2020), and ConvNet (Liu et al., 2022). These target detection algorithms have been widely used in many fields and have gradually become mainstream.

**Figure 1:**
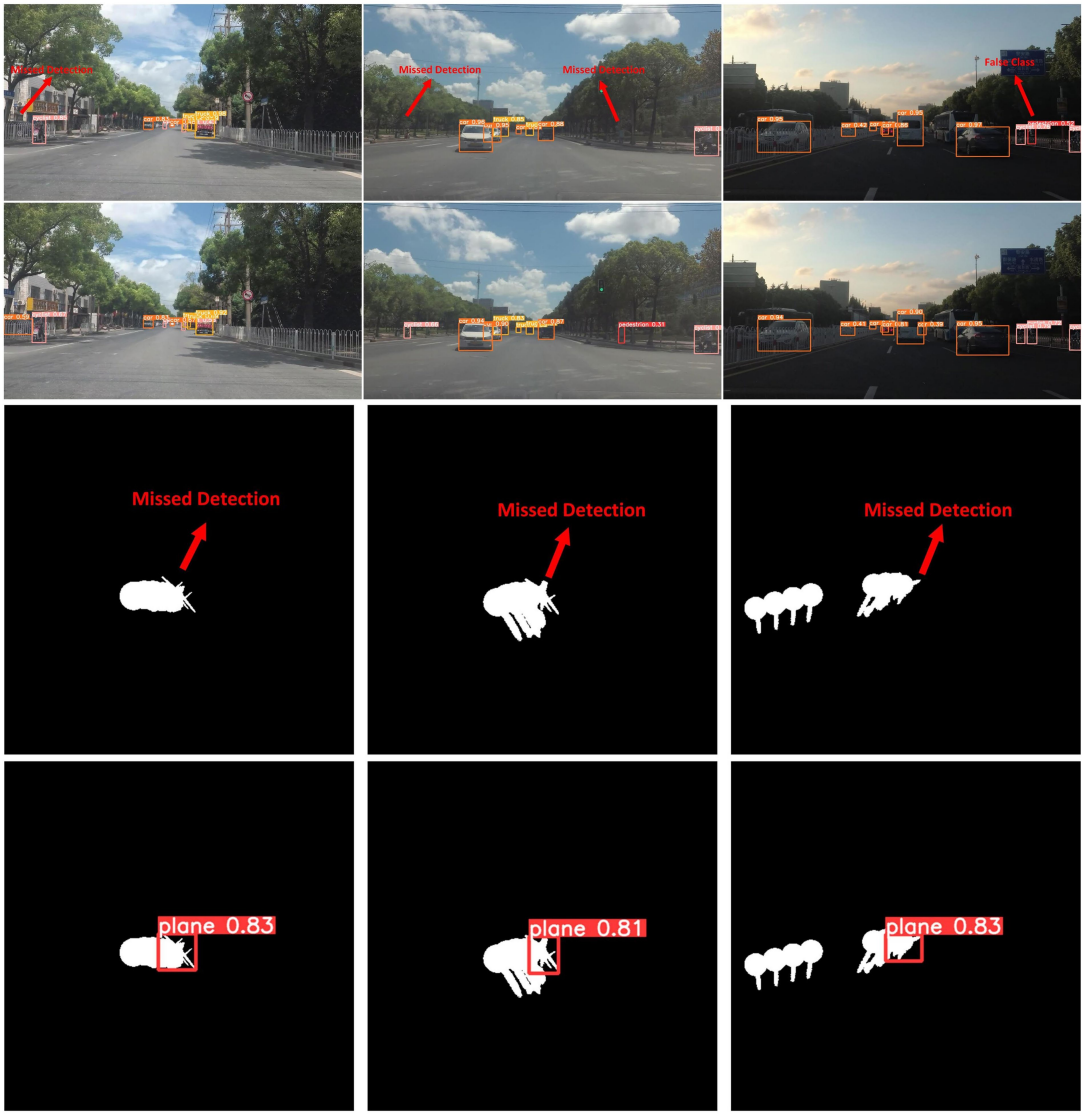
Results on SODA10M dataset and CBIA8K dataset. The first and third rows are the baseline, the second and fourth rows are ours. Our ESF-YOLO is far lower than the baseline in false detections, missed detections, and error categories. At the same time, our ESF-YOLO is much stronger than the baseline in anti-occlusion ability.

**Problem statement:** Common detectors are often designed for specific scenes and objects, and their generalization capabilities and robustness are relatively weak. At the same time, their anti-interference ability is limited when processing images with complex backgrounds, especially images with severe occlusion. These problems often lead to false detections and missed detections, directly affecting algorithm performance. These limitations make these algorithms fall short of expectations when faced with higher diversity and complexity of real-world application scenarios (see Figure 1, first and third rows).

**Motivation:** As an algorithm excellent in both detection performance and running speed, YOLOv5 (Jocher, 2020) has been widely applied in engineering practice. It can achieve fast object detection and has strong robustness, making it suitable for application scenarios that require real-time response. But it still has some design flaws and cannot effectively deal with severe occlusions and complex backgrounds. Therefore, we select YOLOv5 as the baseline and improve it in the hope of improving its detection ability of occlusion maps.

In this study, to address the deficiencies of existing methods, we proposed a new ESF-YOLO (Efficient Scale Fusion YOLO). To validate the efficacy and rationality of these improvements, we have extensively conducted experiments on the SODA10M (Han et al., 2021) dataset and our own CBIA8K dataset (Complex Background Infrared Aircraft Dataset). The experimental results clearly demonstrate that ESF-YOLO has significantly enhanced performance over the YOLOv5 (Jocher, 2020) baseline and has strong anti-occlusion capabilities. The main contributions of this paper are summarized as follows:

1. An innovative Multi-Sampling Conv Module (MSCM) is proposed as the core module of the backbone, which is composed of innovatively designed multi-receptive field CP-Conv operators and feature reuse structures. By making the backbone have different scale sampling rates and repeatedly fused features, the richness of the features extracted from the backbone is greatly improved.
2. A new attention mechanism, Block-wise Channel Attention Module (BCAM), is proposed. BCAM assigns greater weight to effective information-rich patches, making the network pay more attention to the unoccluded parts of the object. It compresses complex background interference and occlusion interference while emphasizing key information in feature maps.
3. A lightweight Decoupled Head (LD-Head) is introduced to solve the coupling problem between the classification branch and the regression branch and avoid excessive increase in calculation amount. While maintaining computational efficiency, it significantly improves detection accuracy.
4. Dynamic confidence loss and adaptive IoU are innovatively designed. Dynamic confidence loss solves the asynchronous problem of IoU labels and predicted values while alleviating the imbalance between positive and negative samples. The adaptive IoU flexibly adjusts the scale factor according to different bounding box sizes, effectively improving regression efficiency for large bounding boxes and reducing positional deviation sensitivity for small ones.

The remainder of the study is organized as follows. Section 2 introduces related work in the field of object detection. Section 3 introduces the overall architecture of ESF-YOLO in detail and elaborates on the proposed targeted improvement strategies. Section 4 performs extensive experimental validation of the proposed improvements. Section 5 is the summary of the full paper.

## 2 Related work

### 2.1 Object detection

Overall, convolutional neural network (Krizhevsky et al., 2017) based detection algorithms can be divided into two main categories: two-stage detectors and single-stage detectors.

#### 2.1.1 Two-stage detectors

The R-CNN (Girshick et al., 2014) can be regarded as the cornerstone work of two-stage detectors. The core idea of R-CNN is to generate a large number of candidate boxes through a sliding window approach. Firstly, R-CNN utilizes methods like selective search to generate numerous region proposals or candidate bounding boxes that may potentially contain the target. Subsequently, for each region proposal, R-CNN employs a Convolutional Neural Network (CNN) to extract relevant features. Finally, a classifier is trained using the extracted features to determine whether each region proposal contains a target of a specific category. If a region is classified as containing the target, R-CNN applies bounding box regression to refine the coordinates of that region’s bounding box, enhancing the accuracy of target localization. However, the design of R-CNN leads to a severe problem of redundant feature recalculations, resulting in significantly low efficiency. Fast R-CNN (Girshick, 2015) introduced a Region of Interest (RoI) pooling layer based on integrating the SPP-Net (He et al., 2015). By utilizing RoI pooling, Fast R-CNN extracts fixed-size features for each proposed region from the CNN feature map. RoI pooling eliminates the need for redundant computations on overlapping regions, thereby improving detection speed to some extent. Despite this enhancement, Fast R-CNN still relies on selective search to obtain candidate regions, and it falls short of addressing the fundamental issues of high computational complexity and slow runtime speed. The subsequent Faster R-CNN (Ren et al., 2017) introduced the Region Proposal Network (RPN) based on Fast R-CNN, significantly reducing the number of candidate regions and thereby improving training and detection speeds. Fast RCNN is considered quite classic in this series of methods. Mask R-CNN (He et al., 2020) added a branch to predict segmentation and replaced the RoI Pooling layer with the RoI Align layer, achieving more precise target identification and being applicable to classification, detection, semantic segmentation, instance segmentation, and other tasks. Cascade R-CNN (Cai and Vasconcelos, 2018) incorporated additional cascaded modules in the subsequent detector part to improve detection performance. Although two-stage detectors achieve high detection accuracy, they are difficult to apply in actual engineering due to their complex structure, slow inference speed, and poor real-time performance.

#### 2.1.2 Single-stage detectors

Single-stage object detection algorithms transform the object localization task into a regression problem. They directly extract features from the input images through a series of convolutional neural networks and predict objects’ categories and locations directly on the generated feature maps. Famous algorithms in this category include the YOLO series (Redmon et al., 2016; Redmon and Farhadi, 2017, 2018; Bochkovskiy et al., 2020; Ge et al., 2021), SSD (Liu et al., 2016), RetinaNet (Lin et al., 2017), MADet (Xie et al., 2023), CANet (Cheng et al., 2023) and more. The key idea of YOLO is to perform object detection through a single forward pass of the neural network, allowing it to predict multiple objects in an image simultaneously. The architecture of YOLO comprises three pivotal components: the backbone, neck, and head. The overall workflow of the network involves the following steps: 1) The backbone network extracts features of varying scales from the input image. 2) The neck network integrates these features of diverse scales. 3) The head network is responsible for predicting the position, category, and confidence. YOLOv5 (Jocher, 2020), as an advanced iteration within the YOLO series, introduces a novel architecture, encompassing the CSPDarknet53 backbone, PANet (Path Aggregation Network) neck, and YOLOv5 head. YOLOX (Ge et al., 2021) proposes a decoupled head based on YOLOv5. By introducing a decoupling head, YOLOX separates classification and regression tasks and uses two networks for training respectively, which significantly improves the object recognition rate. RetinaNet (Lin et al., 2017) uses two simple independent fully connected networks to predict categories and bounding boxes respectively, and proposes a new loss function to solve the imbalance of positive and negative samples. MADet (Xie et al., 2023) draws on the idea of mutual assistance (MA) learning to propose a robust single-stage detector, which solves the problems of feature misalignment and regression inflexibility through feature interactive alignment, mutual assistance regression and quality-oriented loss. CANet (Cheng et al., 2023) is an attention-based image recognition method that improves the discriminative ability of the network by using category-specific dictionary learning to decompose the output of a neural network into category-related features.

Although recent detectors based on Transformer (Vaswani et al., 2017) architectures such as ViT (Dosovitskiy et al., 2021), Swin Transformer (Liu et al., 2021), MyopiaDETR (Li et al., 2023) have demonstrated exceptional performance. However, the high computational cost, memory demands and reliance on large-scale datasets brought by the Transformer (Vaswani et al., 2017) architecture still pose considerable challenges for applying this class of algorithms in practice.

Compared with two-stage detectors, single-stage detectors have the following advantages: 1) The network is trained end-to-end, which simplifies the training process and reduces the complexity of parameter adjustment. 2) Only one forward propagation is needed to complete the detection, which is faster in the inference stage and more in line with actual engineering needs. 3) Smaller targets can be directly predicted, and the application scenarios are wider. As an advanced and highly practical single-stage target detection algorithm, YOLOv5 strikes a balance between accuracy and speed. Therefore, we select it as the baseline for our corresponding study.

### 2.2 Model efficiency

In recent years, in order to better balance computational cost and model accuracy, researchers have proposed many innovative and representative ideas.

One idea is to use lightweight detection models to reduce model complexity as much as possible while ensuring accuracy. To reduce the computational cost, networks typically downsample the input image by a large ratio and perform object detection on small feature maps, such as in ThunderNet (Qin et al., 2019), MobileNet (Howard et al., 2017, 2019; Sandler et al., 2018), LWCDnet (Luo et al., 2022), etc. However, this crude pattern leads models to rapidly downsample in the initial shallow network stages, losing abundant detail information. Thus, the small feature maps in high layers struggle to effectively locate objects’ positional information, which is extremely detrimental to detecting small objects.

Another idea is to introduce an attention mechanism into the network to improve the accuracy as much as possible while ensuring the complexity of the model. By incorporating attention modules, networks can more intelligently select which channels or detail information to focus on, obtaining greater model performance improvement at a smaller computational expense (Klomp et al., 2023). Some common plug-and-play attention modules include SE (Hu et al., 2018), CBAM (Woo et al., 2018), ECA (Wang et al., 2020), ADCM (Guo et al., 2021), etc. By introducing channel attention, the SE module facilitates the model in better understanding the importance of each channel, thereby enhancing the network’s expressive power. This design concept has inspired the subsequent development of attention mechanisms. CBAM combines channel attention with spatial attention, effectively capturing inter-channel relationships and spatial information, thereby improving network performance. As another form of channel attention, ECA differs from SE by adopting one-dimensional local feature statistics instead of global pooling, reducing computational complexity. This makes ECA modules more suitable for resource-constrained scenarios while improving performance. Building upon CBAM, ADCM integrates spatial attention and channel attention with dropout. Channels deemed unnecessary are then removed based on attention magnitude, while crucial channels are retained. Since these modules are independently designed, they can be conveniently embedded into the network without needing to massively modify the overall structure. This design not only maintains the flexibility of the overall model architecture but also provides more powerful context modeling capabilities to improve model performance.

However, these attention mechanisms lack discriminative capabilities in distinguishing between genuine and false information within the feature maps. That is, when an object is occluded, they do not directly lose the features of the occluded part, but regard some features of other objects as that of this object. These attention mechanisms may even give greater weight to these erroneous features. This is obviously not conducive to or even harmful to the effectiveness of the network in extracting features, causing confusion of different object features. To address this problem, we designed a new attention mechanism called BCAM to enhance target-related information and weaken distracting and background information. For partially occluded objects, BCAM enables the network to pay more attention to the effective feature information of the target, greatly reducing background interference and feature confusion between objects.

### 2.3 Loss function

Object detection, as a complex computer vision task, involves both target classification and localization. The design of the loss function must comprehensively consider multiple aspects of task requirements, not only paying attention to classification accuracy but also effectively measuring regression accuracy for localization. Therefore, an appropriate loss function can enable the model to better understand task objectives and more effectively learn target features, thus achieving superior detection performance. Common loss functions in object detection include Smooth L1 Loss (Girshick, 2015), Focal Loss (Lin et al., 2017), IoU Loss (Huang et al., 2017), Balanced Loss (Pang et al., 2019), Generalized Cross Entropy Loss (Kurian et al., 2021) and more. Focal Loss is a loss function designed to address the class imbalance problem in object detection tasks. The key idea behind Focal Loss is to down-weight the contribution of well-classified examples, focusing more on the hard-to-classify examples, particularly those from the minority class. This is achieved by introducing a modulating factor that reduces the loss for well-classified examples. Balanced Loss is designed to deal with the category imbalance problem and ensure that the contribution of each category is balanced when training the model. Balanced Loss prevents the model from being too biased towards categories that appear more frequently, thereby increasing its sensitivity to categories that appear less frequently and ensuring that the model can receive appropriate attention to samples of different categories during the training process.

As an anchor-based detector, YOLOv5 generates a large number of predefined anchors with different scales and aspect ratios on the image to cover various target shapes and sizes. However, since the number of anchors far exceeds the number of targets, an extremely imbalanced assignment of positive and negative samples occurs. Although Focal Loss and Balanced Loss reduce the weight of easy examples to help networks focus more on hard examples, they still cannot fundamentally resolve sample imbalance. In response to the YOLOv5 loss function defects, we propose the Dynamic Loss and adaptive IoU to significantly optimize the loss function design.

## 3 Method

**Figure 2:**
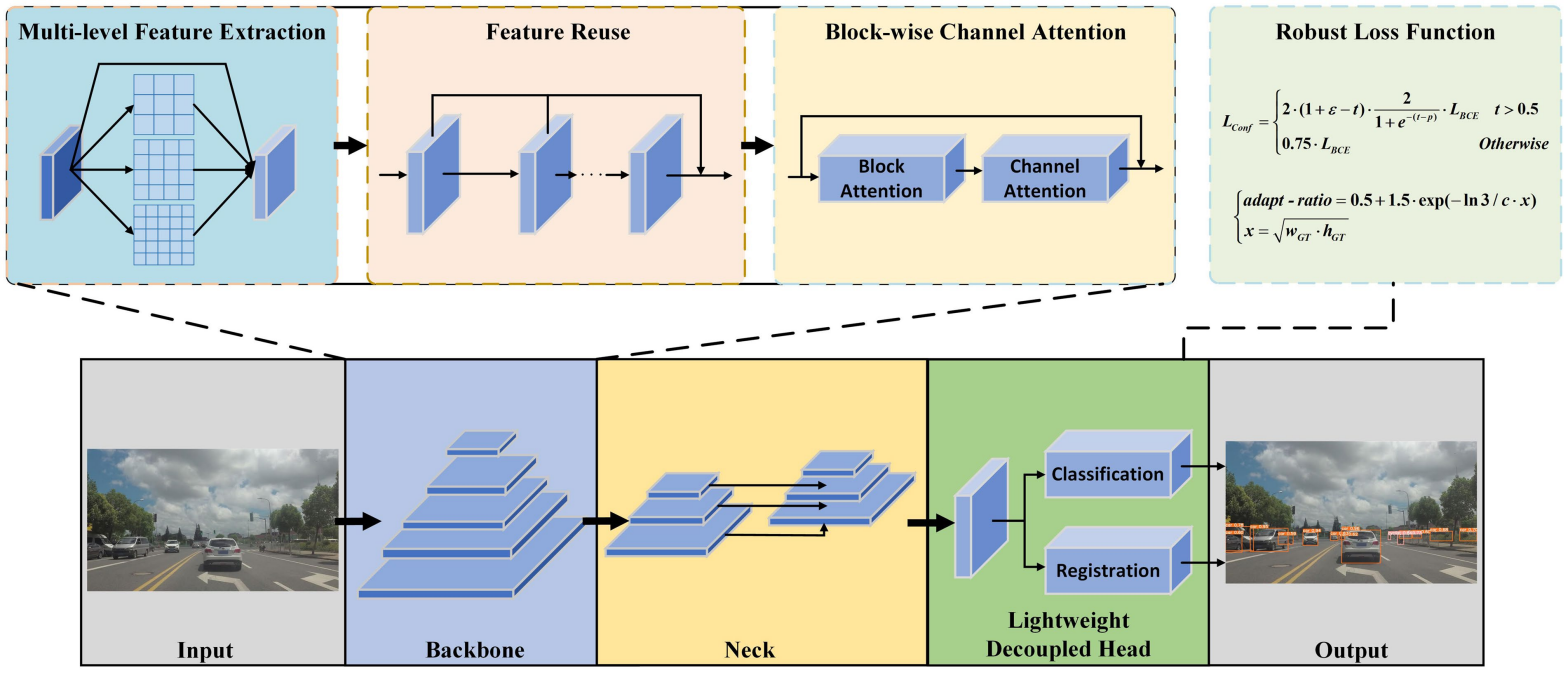
The overall structure of ESF-YOLO.

This section provides a detailed overview of the implementation details of ESF-YOLO. First, to enhance the feature fusion capability of the backbone, the Multi-Sampling Conv Module is introduced. This module consists of CP-Conv operators and feature reuse mechanisms, serving as the fundamental module of the backbone network. Second, in order to improve the detector’s ability to detect severely occluded objects, a novel Block-wise Channel Attention Module is introduced in the backbone network and placed after each MSCM module. This module focuses on the effective information and contextual information of the unoccluded parts of the object. Third, in view of the problem that classification and positioning tasks should not be coupled, and the traditional decoupled head requires too much calculation, an efficient lightweight Decoupled Head is redesigned. Fourth, in order to overcome the shortcomings of the loss function in the original YOLOv5, a dynamic loss function and adaptive IoU are proposed. The combination of these innovative designs enables ESF-YOLO to outperform baseline models comprehensively in object detection tasks. The overall structure of ESF-YOLO is illustrated in Figure 2.

### 3.1 Multi-level feature extraction

#### 3.1.1 CP-Conv operator

**Figure 3:**
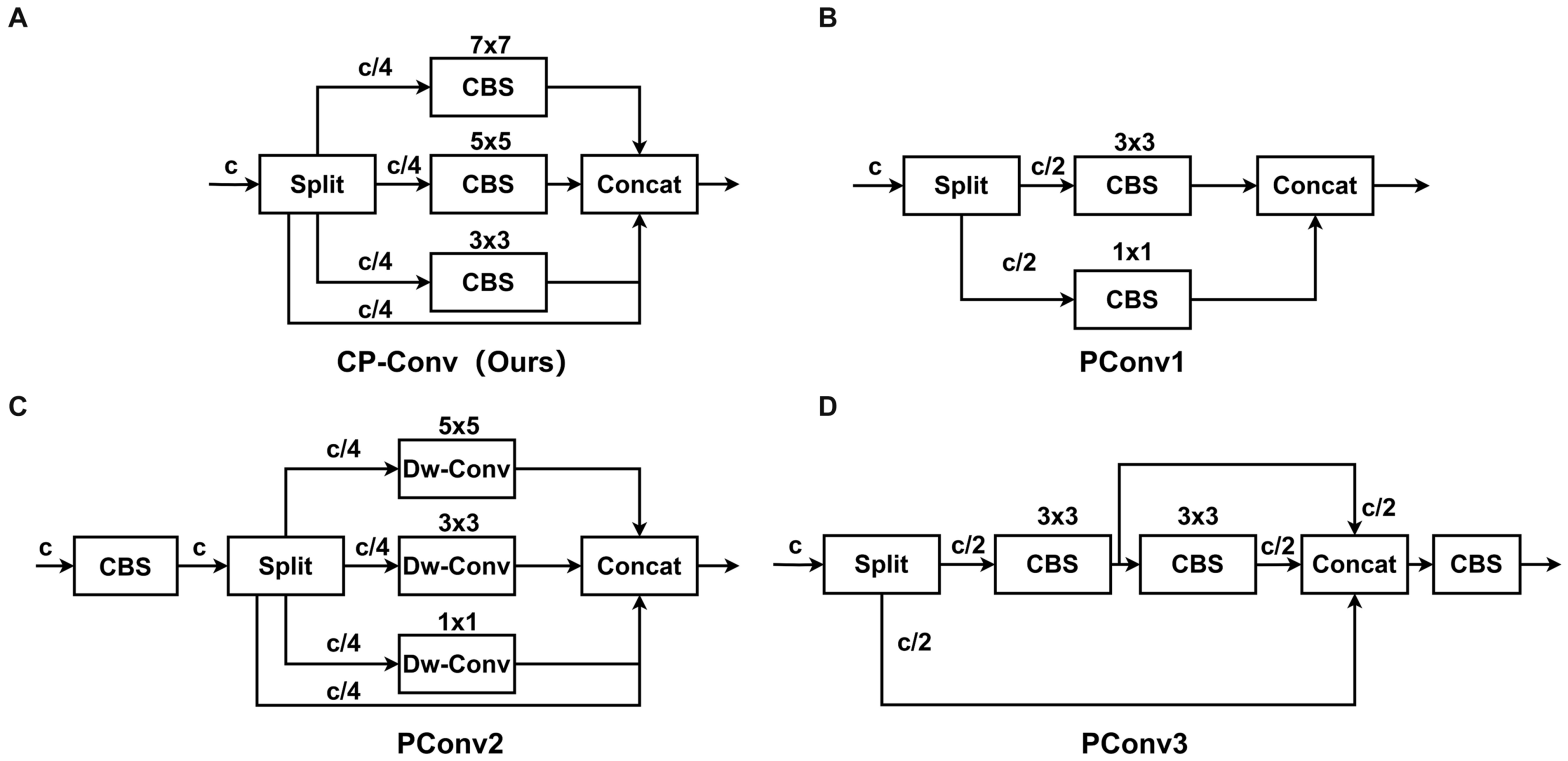
Structure diagrams of CP-Conv and common PConv. Our proposed CP-Conv **(A)**. PConv1 **(B)**. PConv2 **(C)**. PConv3 **(D)**.

Inspired by the Partial Convolution (P-Conv) operator proposed in FasterNet (Chen et al., 2023), we redesign the CP-Conv operator by constructing a multi-scale receptive field structure to more effectively extract spatial features. The schematic diagram of the CP-Conv operator is illustrated in Figure 3A. The CP-Conv operator first divides the feature map into four parts in the channel dimension. Three parts, respectively, apply convolutions with kernel sizes of 3 × 3, 5 × 5, and 7 × 7. Finally, the outputs are concatenated in the channel dimension. The receptive field sizes of the four branches are 1 × 1, 3 × 3, 5 × 5, and 7 × 7 respectively, which enriches the diversity of receptive fields and facilitates extracting spatial features. Combining the CP-Conv operator and residual connection, the basic building block of MSCM, CP-Block, is obtained.

The newly designed CP-Conv enables the neural network to capture the information of the input data at different scales. By using receptive fields of different sizes at different levels of the network, the network can pay attention to both local and global features to obtain richer contextual information. At the same time, since the objects in many images have different scales, or the objects in the images are deformed, rotated, or scaled. By using multiple receptive fields, neural networks can better understand and process the hierarchical structure of these objects, adapting small-scale details to large-scale overall shapes. CP-Conv can improve the network’s resistance to deformation and enable the network to better adapt to changes at different scales. In general, CP-Block has far fewer parameters than the residual module in C3, and its perceptual capabilities are much stronger than the residual module in C3, achieving a win-win situation of small calculation amount and high performance.

#### 3.1.2 Feature reuse

**Figure 4:**
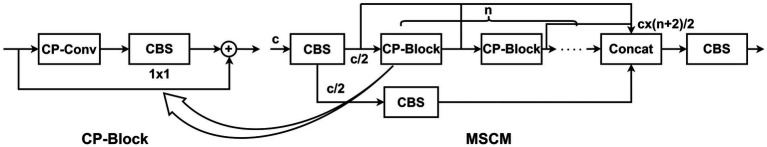
Structural diagram of MSCM. MSCM consists of n cascaded CP-Blocks, with feature fusion applied to the outputs of all CP-Blocks.

In the evolution of convolutional neural networks, researchers have continuously explored their architectures. DenseNet (Huang et al., 2017) adopts dense connections to continually aggregate network features, but the redundant aggregation of the features has led to significant memory occupancy and unnecessary computational burden. To improve this redundant feature aggregation, VoVNet (Lee et al., 2019) proposed One-Shot Aggregation (OSA). This method eliminates many connection branches and only performs operations on the features before the final aggregation layer. As illustrated in Figure 4, to efficiently reuse information from the intermediate feature maps, we repeat the CP-Block modules n times in MSCM. For the input feature map, we first evenly split it into two parts along the channel dimension, and obtain two C/2 channel feature maps through two 1 × 1 convolution modules. Next, we input one feature map into the aggregation branch. After each CP-Block module, we aggregate the result with the original input feature map of this branch. Then, we concatenate the feature outputs of the two branches along the channel dimension to form a feature map with C × (*n* + 2)/2 channels. Finally, we adjust the number of channels to C through a convolution module to ensure the channel number remains unchanged. Through feature reuse, the information learned at the lower level can be passed to higher levels, allowing the network to utilize the detailed information of the underlying features for deeper understanding and learning. At the same time, the features learned by the feature reuse structure are more universal, which helps to combat over-fitting and enable the model to better generalize to unseen data. In terms of efficiency, feature reuse can reduce the number of parameters that need to be trained in the model, thereby reducing the complexity of the model.

### 3.2 Block-wise channel attention

The human visual system receives a tremendous amount of sensory inputs, far exceeding what the brain is able to fully process. However, not all stimuli have an equal influence. The convergence of consciousness and focus enables the brain to shift attention toward objects-of-interest amid complex visual environments. Inspired by this observation, attention mechanisms have been introduced into computer vision (Guo et al., 2022). Attention mechanisms empower convolutional neural networks to concentrate on informative cues while suppressing unnecessary ones by dynamically recalibrating weights based on input features (van Dyck et al., 2022).

The bounding box finally predicted by the network is a rectangle, and the label of the bounding box in the dataset is also a rectangle. During the training process, the backbone network is only responsible for extracting features and does not have the ability to judge the effectiveness of features. The backbone network may regard some other interference as effective features of the target. That is, there will be the following two problems: 1) When the environmental background of the input image is complex, the network may misjudge the background features as the object features; 2) When an object is partially occluded, the network may regard the features of other objects displayed in front as the features of the object. Common attention mechanisms treat every pixel in the feature map equally and obtain the spatial importance of features through the relationship between pixels. This will lead to a serious problem: when receiving these features, the attention mechanism will mistakenly believe that they are equally important, and even give greater weight to invalid features, thus seriously affecting the accuracy of network prediction.

In order to eliminate the interference of negative features, BCAM is proposed. By modeling the feature maps of each channel independently, BCAM can perform targeted individual analysis of the feature maps of different channels. On this basis, BCAM uses large-size convolution kernels to sample each channel, abandoning the traditional attention mechanism’s strategy of over-considering the microscopic relationships between pixels. On the contrary, BCAM emphasizes the importance of “spatial blocks” in the feature map to highlight the relatively important parts of the spatial domain, that is, the effective features of the object. Secondly, a pooling operation is applied to extract these effective features from “spatial blocks” and convert them into “spatial points” features to reduce the feature map size and reduce computational complexity. Then, BCAM obtains channel importance and assigns weights, and performs channel fusion through fully connected layers. Finally, BCAM fuses the channel importance and spatial importance of all feature maps obtained. BCAM greatly enhances the network’s ability to distinguish effective features.

**Figure 5:**
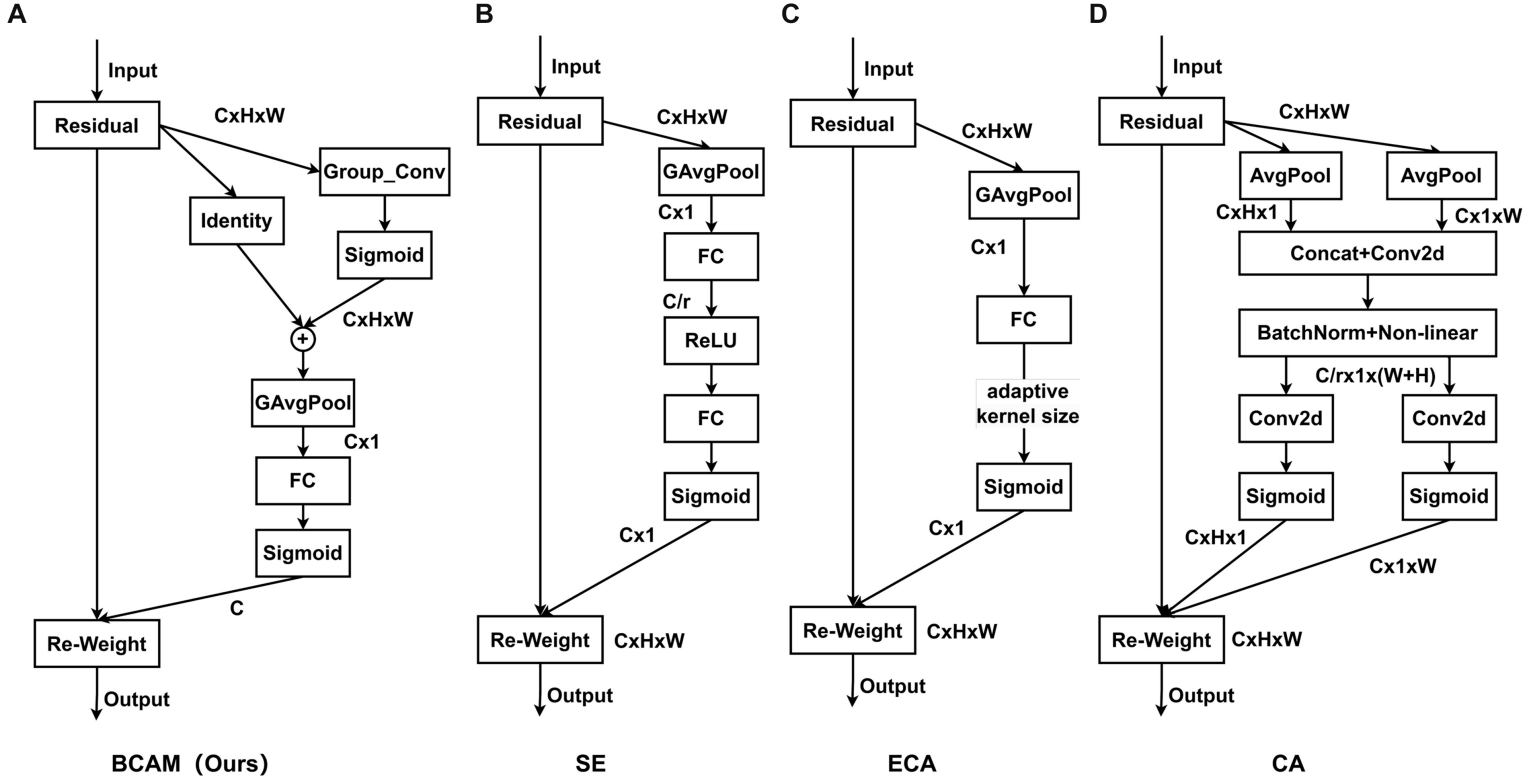
Structural diagrams of BCAM and common attention modules. Our proposed BCAM **(A)**. SE **(B)**. ECA **(C)**. CA **(D)**.

The structure of BCAM is illustrated in Figure 5A, consisting of four steps: effective feature extraction, feature calibration, channel reconstruction, and feature fusion. First, we apply convolutions with large kernel sizes to highlight effective features, suppress invalid features, and obtain the importance of spatial locations, allowing the network to focus on more critical effective regions:


(1) $$\left\{ \begin{array}{l} F^{1}=G\mathrm{roup}:\mathrm{Conv}\left(F\right)\\ F^{2}=\mathrm{Sigmoid}\left(F^{1}\right) \end{array}\right.$$

Then, we fuse the “spatial blocks” features with the original features and use average pooling to obtain the “spatial points” features:


(2) $$F^{3}=\mathrm{AvgPool}\left(F^{2}+F\right)$$

Afterwards, we utilize a fully connected layer to reconstruct the channel features and obtain the channel-level importance of all feature maps:


(3) $$F^{4}=\sigma \left(\mathrm{Linear}\left(F^{3}\right)\right)$$

Finally, we multiply the attention weights element-wise with the original features to get the final output:


(4) $$F_{\mathrm{final}}=F^{4}\times F$$

### 3.3 Lightweight Decoupled Head

In object detection, there is a conflict between classification and regression tasks, and it is unreasonable to directly use simple convolutional layers to generate prediction outputs. Classification and regression are used for identification and positioning respectively, and the features of concern during feature learning are different. The output result of classification is discrete and is the category to which the object belongs. The output result of regression is continuous, which is the value of the object’s position, changing within a range. YOLOv5 uses coupling heads so that the two branches share most of the parameters, which limits the performance of target detection to a certain extent. In YOLOX (Ge et al., 2021), the authors introduced a decoupled head to replace the original detection head to decouple the positioning and classification tasks. Two different sub-networks are used to handle localization and classification tasks respectively, achieving significant performance improvements. However, since the detection head usually needs to process the prediction outputs from different stages of the neck network, the introduction of complex decoupling heads incurs huge time costs during execution.

**Figure 6:**
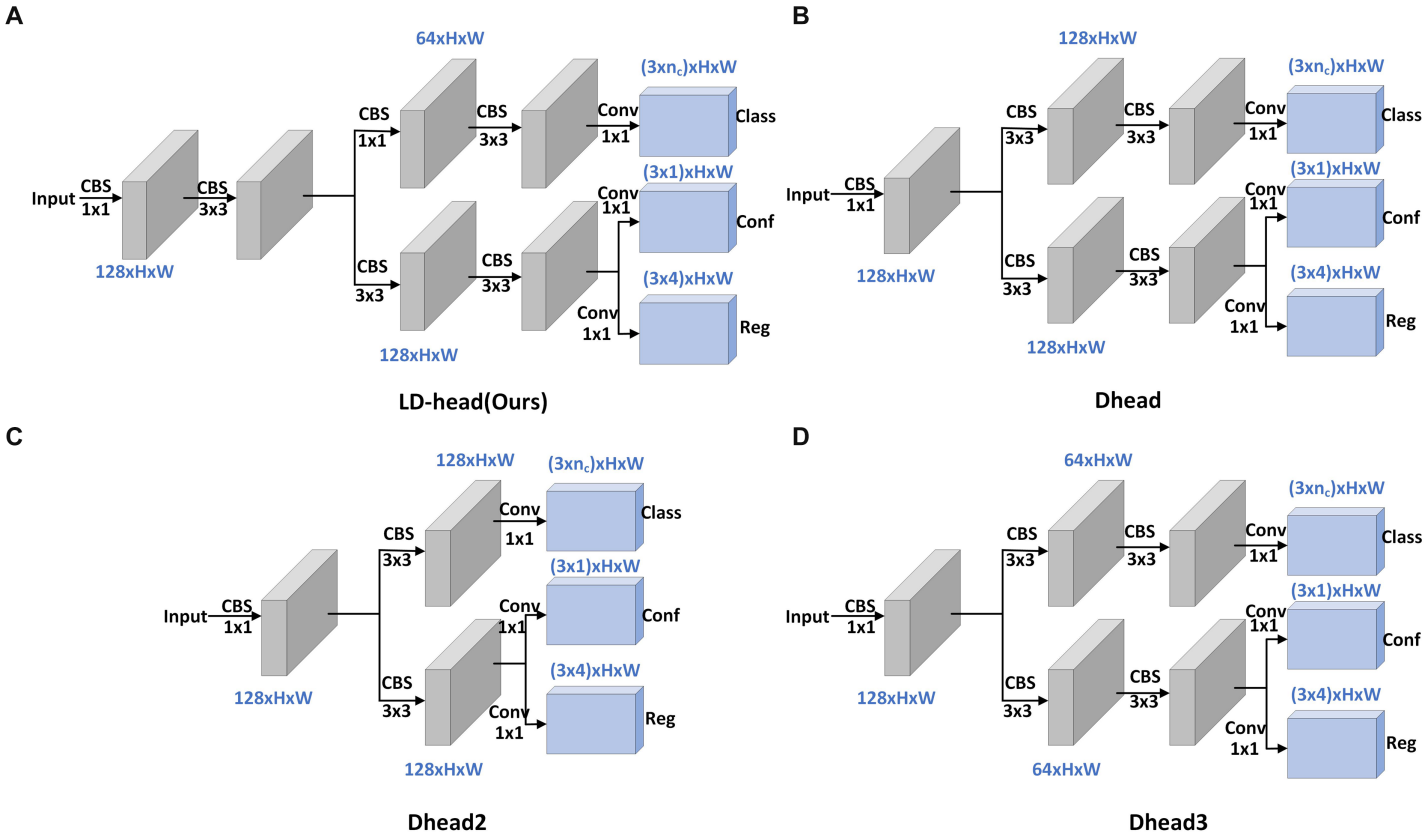
Structural diagrams of LD-Head and common decoupled head variants. Our proposed LD-Head **(A)**. Dhead **(B)**. Dhead2 **(C)**. Dhead3 **(D)**.

The decoupled head can speed up model convergence and improve detection accuracy, but it will bring additional parameters and calculation costs. The network expects a detection head with high accuracy and low computational load. In order to solve the problem of large amount of calculation and parameters of the decoupled head, we propose a lightweight Decoupled Head, called LD-Head. Considering that the input feature maps of detection heads have at least 128 channels, and these feature maps are for detecting small objects, reducing the number of channels of feature maps in the main path of detection head is thus inadvisable. To more fully utilize the information in feature maps, 3 × 3 convolution modules are additionally introduced in the main path. Compared with localization, the classification task is relatively simple. Therefore, we have redesigned the structure of the classification branch. Specifically, the first 3 × 3 convolution module in the classification branch is replaced with a 1 × 1 convolution block, while simultaneously reducing the number of channels of the feature maps to decrease computational and parameter complexity. The structures of the LD-Head and other decoupled head variants are illustrated in Figure 6.

### 3.4 Robust loss function design

#### 3.4.1 Dynamic confidence loss

The loss function consists of weighted classification loss, confidence loss and regression loss. Among these three parts, the confidence loss accounts for the largest proportion and involves calculations over all positive and negative samples. Specifically, the IoU value is utilized to determine positive sample labels when computing confidence loss. However, through analyzing loss curves over positive samples under different label values, we discover this setting may hinder effective regression for high-quality positive samples.

**Figure 7:**
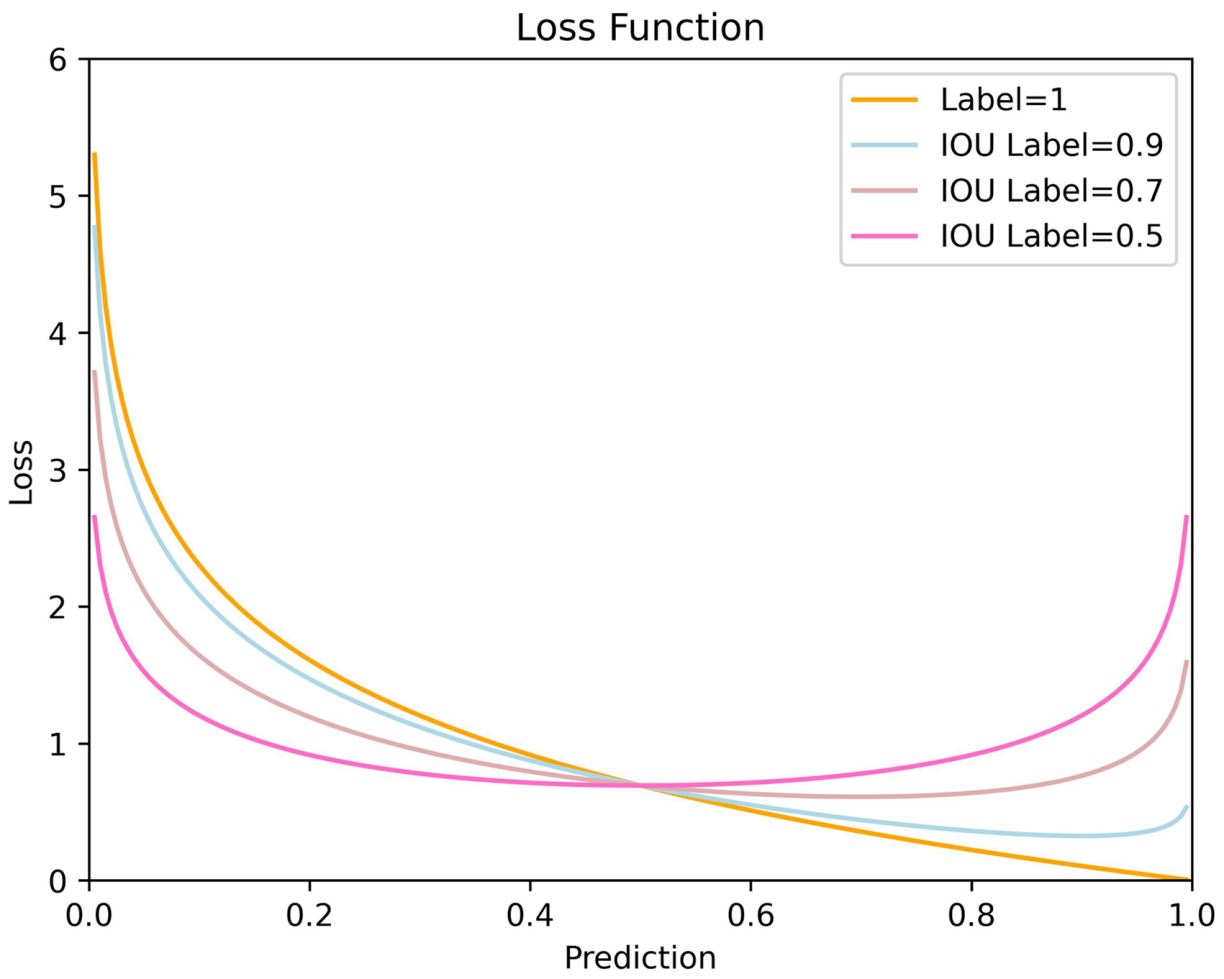
Loss curve with respect to prediction for different label values. It is evident that when the predicted values are relatively high, the smaller the IoU label value, the more unfavorable it is for confidence training. This may even hinder the optimization of confidence.

The network expects confidence to increase as IoU grows, reflecting higher-quality detection results. However, as training confidence is easier than localization, IoU and predicted confidence struggle to grow synchronously. In fact, since the IoU value is always less than 1 during training, positive sample labels are persistently below 1 as well, which leads to a problem. That is when predictions approach 1, the loss will instead enlarge, especially when IoU labels are small, as shown in Figure 7. This reflects defects in the loss function design, not achieving the effects we expect.

We expect that:1) As confidence increases, the loss function should decrease; 2) Larger IoU label values should correspond to smaller losses; 3) When predictions surpass labels, the loss should still diminish. We design a new confidence loss function that meets the above expectations. The new confidence loss function is:


(5) $$L_{\mathrm{Conf}}=\{\begin{array}{ll} 2\cdot \left(1+\varepsilon -t\right)\cdot \frac{2}{1+e^{-\left(t-p\right)}}\cdot L_{BCE} & t>0.5\\ 0.75\cdot L_{BCE} & \mathrm{Otherwise} \end{array}$$

where $\varepsilon =e^{-3}$, t is the label value, p is the predicted value, and $L_{BCE}$ is the Binary Cross-Entropy Loss.

Specifically, when the label value is relatively large (t>0.5), by introducing a dynamic coefficient, the loss is increased; when the label value is relatively small (t<0.5), by introducing a coefficient less than 1, the loss is decreased. This measure achieves a balance between positive and negative samples. When the label value is relatively large (t>0.5), we directly incorporate the label value into the loss function calculation. As the label value increases, the loss for positive samples decreases. This measure effectively addresses the asynchronous issue between IoU labels and confidence predictions.

#### 3.4.2 Adaptive IoU

**Figure 8:**
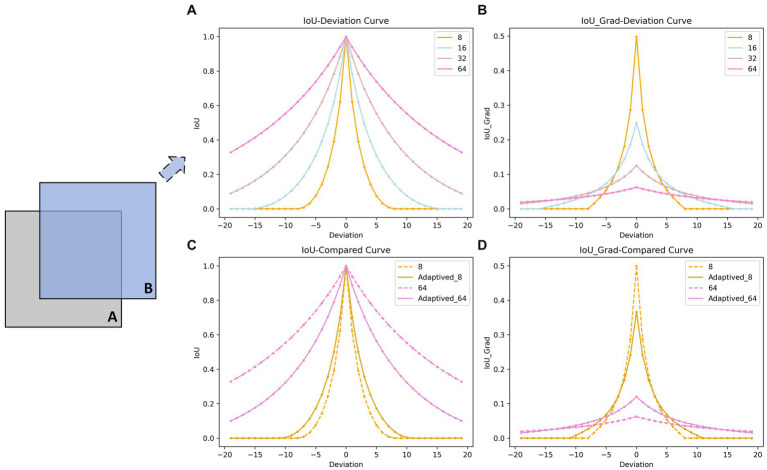
Comparison of IoU-Deviation curves **(A)** and Gradient-Deviation curves **(B)** for different-scale bounding boxes. Comparison of IoU-Deviation curves **(C)** and Gradient-Deviation curves **(D)** before and after adding adapt-ratio. The bounding box B moves along the diagonal away from bounding box A. The horizontal axis represents the deviation, while the vertical axis represents the corresponding IoU/gradient values. Our approach accelerates the regression of large bounding boxes while reducing the position sensitivity of small bounding boxes (the real lines).

Bounding box prediction plays a crucial role in object detection tasks. The computation and optimization of box IoU not only enables more accurate predictions from the regression branch, but also provides high-quality positive sample labels for the confidence branch. As illustrated in Figure 8, we maintain boxes A and B at the same size, with B shifted diagonally away from A. Figures 8A,B exhibit IoU values and gradient curves under four different sizes (8, 16, 32, 64) of boxes. The horizontal axis denotes pixel deviation while the vertical axis represents IoU/gradient. It can be observed that smaller boxes are more sensitive to positional deviations. When the deviation between A and B becomes too large, both IoU and gradient values become 0, causing the regression branch to lose gradients for further optimization. Although larger boxes are more robust to positional deviations, their gradients are generally smaller overall, leading to a slower optimization speed and lower efficiency for the regression branch.

It can be concluded that: For small boxes, setting larger artificial bounds helps alleviate sensitivity to deviations; For large boxes, smaller artificial bounds can increase IoU gradients to accelerate optimization efficiency. We have innovatively designed an adaptive scale factor to automatically tailor the artificial bound size to accommodate boxes of various dimensions in datasets. The adaptive ratio is:


(6) $$\mathrm{adapt}-\mathrm{ratio}=0.5+1.5\cdot \exp\left(-\ln3/c\cdot x\right),x=\sqrt{w_{GT}\cdot h_{GT}}$$

Figures 8C,D illustrate the curves of IoU and its gradient with respect to deviation for typical large-sized boxes (64) and small-sized boxes (8) after adjusting for adaptive scale factors. The optimization of adaptive scale factors enhances the regression performance of both sizes of the boxes. Specifically, this optimization process not only strengthens the robustness of small-sized boxes but also expedites the regression process for large-sized boxes.

## 4 Experiments and analysis

### 4.1 Datasets

We conducted experiments on the SODA10M (Han et al., 2021) dataset and our self-constructed CBIA8K dataset to comprehensively evaluate the proposed method. For object detector designed for complex scenes, we specifically emphasize their applicability across various scenarios.

#### 4.1.1 SODA10M

SODA10M covers a variety of different road scenes, taking into account diverse weather conditions and conducting data collection during various time periods, including daytime, nighttime, early morning, and dusk. The dataset is annotated with six primary scene categories (Pedestrian, Cyclist, Car, Truck, Tram, Tricycle). The objects in the SODA10M dataset have serious mutual occlusion. While the SODA10M dataset comprises a total of 10,000 annotated images, the distribution of categories is uneven, with limited images containing Tram and Tricycle categories. Therefore, we opted to focus on four categories with a more balanced distribution for further investigation: Pedestrian, Cyclist, Car, and Truck. For experimentation purposes, we randomly split the data into training and testing sets with a ratio of 7:3.

#### 4.1.2 CBIA8K

CBIA8K is a challenging infrared aircraft dataset generated through simulation. This dataset exclusively comprises a single target category, namely aircraft, yet it features complex background interference such as decoy flares, causing severe occlusion. CBIA8K consists of a total of 8,000 annotated images, and the data is randomly partitioned into training and testing sets with a ratio of 2:8.

### 4.2 Evaluation metrics

In our experiments, we employed the widely used evaluation metrics for object detection, namely the Average Precision series (AP), including AP50, AP75, and mAP. Average Precision is a commonly used metric to evaluate the performance of object detection models. In object detection tasks, AP represents the average precision of the model across different categories. The calculation of AP involves computing precision and recall for each category and then plotting the Precision-Recall Curve. AP is the area under this curve, indicating the average performance of the model at different recall levels. In summary, AP considers the model’s performance across different categories and provides a more holistic evaluation through the analysis of precision and recall. The formula for computing AP is:


(7) $$\left\{ \begin{array}{l} AP=\overset{1}{\underset{0}{\int }}P\left(r\right)dr\\ \mathrm{mAP}=\frac{1}{N}\overset{N}{\underset{i=1}{\sum }}AP_{i} \end{array}\right.$$

Additionally, to comprehensively assess and quantify model efficiency, we also introduced Parameters, Giga Floating-point Operations Per Second (GFLOPs) and Frames Per Second (FPS) as evaluation criteria. Parameters refer to the total number of weights and biases that need to be learned in a neural network. More parameters typically indicate a more complex model. GFLOPs represents the number of floating-point operations that a model performs in 1 second. It is a metric used to assess the computational complexity and performance of a model. In the field of deep learning, researchers often pay attention to these two metrics to evaluate the size, computational requirements, and performance of models. Smaller numbers of parameters and GFLOPs typically imply a more lightweight model with higher computational efficiency, which can be suitable for embedded devices or environments with resource constraints. FPS typically refers to the number of frames processed per second by a model, representing the quantity of images the model can detect and recognize within 1 second. It is an indicator to measure the real-time performance of the model.

### 4.3 Training details

We choose YOLOv5s as the baseline for our experiments and utilize four NVIDIA 4090 GPUs. The initial learning rate is set to 0.01, employing a cosine annealing learning rate strategy. The training process utilizes the SGD optimizer for 300 epochs with a momentum value set to the default of 0.937. Additionally, we configure the batch size to be 128.

### 4.4 Comparative experiments

#### 4.4.1 Operator selection

Figure 3A illustrates the proposed CP-Conv operator, while Figures 3B–D showcase several common operators. As shown in Table 1, we conducted comparative experiments involving various operators. Our CP-Conv operator exhibits a rich receptive field and powerful feature fusion capabilities, outperforming the baseline in all aspects while maintaining lower parameter counts and GFLOPs. Specifically, on the SODA10M dataset, the CP-Conv operator demonstrated improvements of 1.09% in AP50, 0.7% in AP75, and 0.69% in mAP compared to the baseline. Notably, while achieving performance comparable to PConv3, the CP-Conv operator boasts significantly lower parameter counts and GFLOPs. Compared with PConv1 and PConv2, the calculation amount is similar, but the accuracy is much higher than them. CP-Conv achieves a perfect balance between performance and computational effort. On the CBIA8K dataset, despite performance declines observed with other operators, the CP-Conv operator still achieved a 0.61% increase in AP50. Compared with the baseline, while performance is improved, Params are reduced by 10.3% and GFLOPs are reduced by 13.9%.

**Table 1:** Comparison results of CP-Conv (ours) with other common convolutional operators.

Dataset	Operator	AP50	AP75	mAP	Params	GFLOPs
SODA10M	Baseline	67.98	43.13	42.38	7.02	15.8
	PConv1	68.71	43.44	42.73	6.34	13.8
	PConv2	68.44	43.5	42.74	6.17	13.3
	PConv3	69.49	44.34	43.57	6.77	15
	CP-Conv (ours)	69.07	43.83	43.07	6.3	13.6
CBIA8K	Baseline	92.06	78.78	67.44	7.01	15.8
	PConv1	92.47	76.17	65.52	6.33	13.8
	PConv2	92.33	77.15	66.31	6.16	13.3
	PConv3	92.76	76.52	67.03	6.77	15
	CP-Conv (ours)	92.67	78.31	67.47	6.29	13.6

#### 4.4.2 Configuration of “*n*”

To determine the optimal configuration for parameter “*n*,” a series of experiments were conducted, and the results are presented in Table 2. The experimental findings indicate that increasing the number of “*n*” in each MSCM results in a significant increase in computational and parameter overhead, with minimal corresponding performance gains. Consequently, in order to achieve the maximum benefit, that is, the balance between speed and accuracy, we ultimately selected the configuration for parameter “*n*” as 1, 2, 3, 1.

**Table 2:** Comparison results of the parameter “*n*” under different configuration.

Dataset	Operator	AP50	AP75	mAP	Params	GFLOPs
SODA10M	1,2,3,1	68.87	44.06	42.97	6.42	14.1
	1,2,8,2	68.81	44.06	43.06	7.18	15.7
	3,3,9,1	68.91	44.35	43.15	6.96	16.6
CBIA8K	1,2,3,1	92.80	76.57	66.26	6.41	14
	1,2,8,2	92.89	77.16	66.59	7.17	15.7
	2,2,8,1	92.57	78.00	66.53	6.84	15.7
	3,3,6,2	92.29	75.98	65.91	7.04	16

#### 4.4.3 Attention mechanism test

In Figure 5A, we illustrate our proposed BCAM, while Figures 5B–D showcase SE (Woo et al., 2018), ECA (Wang et al., 2020), and CA (Hou et al., 2021) modules, respectively. BCAM is designed to focus the network more on bottom-up details, effectively locating specific feature positions, and allocating larger weights to channels with more local information. This significantly enhances the network’s feature fusion and anti-interference capabilities. As shown in Table 3, whether it is SE, CA, ECA, or our BCAM, none of them introduce additional computational burden. However, SE, CA, and ECA exhibit a performance decline trend on a specific dataset, while BCAM demonstrates broader applicability and achieves significant performance improvement on both datasets. Specifically, on the SODA10M dataset, BCAM increases AP50 by 0.36%, and on the CBIA8K dataset, it enhances AP50 by 0.62%.

**Table 3:** Comparison results of BCAM (ours) and other common attention mechanisms.

Dataset	Operator	AP50	AP75	mAP	Params	GFLOPs
SODA10M	Baseline	69.14	43.84	43.13	6.92	15.5
	SE	68.45	43.59	42.81	6.96	15.5
	ECA	69.22	44.16	43.20	6.92	15.5
	CA	68.78	43.82	42.99	6.99	15.6
	BCAM (ours)	69.50	43.88	43.12	6.99	15.7
CBIA8K	Baseline	92.96	78.74	68.20	6.91	15.5
	SE	93.05	78.82	68.00	6.96	15.5
	ECA	93.13	78.86	67.84	6.91	15.5
	CA	92.81	77.38	65.83	6.98	15.6
	BCAM (ours)	93.58	80.11	68.23	6.98	15.7

#### 4.4.4 Decoupled head selection

Figure 6A depicts our proposed LD-Head, while Figures 6B–D showcase various variants of the decoupled heads. As indicated in Table 4, our LD-Head demonstrates superior performance on both the SODA10M and CBIA8K datasets. Moreover, compared to the decoupled head (Dhead) proposed in YOLOX (Ge et al., 2021), our LD-Head exhibits reduced computational complexity and better compatibility with the output features from the neck network. Specifically, Params are reduced by 3.5% and GFLOPs are reduced by 5.5%. LD-Head improves accuracy while reducing the amount of calculation, ensuring the real-time requirements of the detector.

**Table 4:** Comparison results of LD-Head (ours) and other common decoupled head variants.

Dataset	Operator	AP50	AP75	mAP	Params	GFLOPs
SODA10M	Dhead	70.63	46.67	45.30	8.86	30.9
	Dhead2	69.75	45.59	44.35	7.97	23.5
	Dhead3	70.08	45.69	44.67	7.75	21.6
	LD-Head (ours)	70.89	46.49	45.36	8.55	29.2
CBIA8K	Dhead	93.84	78.08	68.09	8.86	30.9
	Dhead2	92.89	79.32	68.44	7.97	23.5
	Dhead3	93.32	78.94	69.05	7.74	21.6
	LD-Head(ours)	93.71	78.49	68.52	8.55	29.2

#### 4.4.5 Loss function test

Table 5 illustrates a comparative analysis of the results between our proposed Dynamic Loss and commonly used BCE Loss and Focal Loss. The Dynamic Loss effectively addresses deficiencies in loss function design and mitigates the imbalance between positive and negative samples. Its performance surpasses that of other loss functions comprehensively, particularly for the CBIA8K dataset, with an impressive increase of 2.68% in AP75 and a 1.54% improvement in mAP.

**Table 5:** Comparison results of dynamic loss (ours) and other common loss functions.

Dataset	Operator	AP50	AP75	mAP	Params	GFLOPs
SODA10M	BCE loss	70.89	46.49	45.36	8.55	29.2
	Focal loss	70.12	45.98	44.93	8.55	29.2
	Dynamic loss (ours)	71.10	47.26	45.78	8.55	29.2
CBIA8K	BCE	93.71	78.49	68.52	8.55	29.2
	Focal loss	91.07	75.55	65.00	8.55	29.2
	Dynamic loss (ours)	93.24	81.17	70.06	8.55	29.2

#### 4.4.6 Coefficient “ratio” analysis

To demonstrate the substantial advantages of our proposed adaptive ratio, we compared adapt-ratio with static ratios (set to 0.8, 0.9, 1.0, 1.1, and 1.2, respectively). The experimental results are detailed in Table 6. Due to the inability of static ratios to simultaneously cater to both large and small bounding boxes, their performance improvement over the baseline is considerably limited. In contrast, our adaptive ratio autonomously adjusts based on the bounding box size, accommodating the requirements of different-sized bounding boxes. On the SODA10M dataset, adapt-ratio shows an improvement of 0.81% in AP50, 0.64% in AP75, and 0.6% in mAP. On the CBIA8K dataset, there is an enhancement of 1.06% in AP50, 2.43% in AP75, and 2.77% in mAP.

**Table 6:** Comparison results of adapt-ratio (ours) and other static ratios (0.8–1.2).

Dataset	Operator	AP50	AP75	mAP	Params	GFLOPs
SODA10M	0.8	71.14	47.30	45.84	8.55	29.2
	0.9	71.432	47.70	46.13	8.55	29.2
	1.0(baseline)	71.10	47.26	45.78	8.55	29.2
	1.1	70.52	46.46	45.31	8.55	29.2
	1.2	70.13	47.00	45.21	8.55	29.2
	Adapt-ratio (ours)	71.91	47.90	46.38	8.55	29.2
CBIA8K	0.8	93.81	82.64	71.84	8.55	29.2
	0.9	94.19	82.51	71.68	8.55	29.2
	1.0(baseline)	93.24	81.17	70.06	8.55	29.2
	1.1	93.53	79.7	69.37	8.55	29.2
	1.2	93.77	81.25	69.72	8.55	29.2
	Adapt-ratio (ours)	94.30	83.60	72.83	8.55	29.2

### 4.5 Ablation studies

To verify the absence of conflicts among the proposed improvements and demonstrate their cumulative performance enhancement, we conducted comprehensive ablation experiments on the SODA10M and CBIA8K datasets. The experiment adopts the control variable method and is conducted according to the ablation experimental paradigm. In order to ensure the authenticity of the experimental data and avoid accidental errors, we repeated the experiment several times and took the average value for each additional module. Using the initial unimproved detector as the baseline, improved modules are gradually added. The detailed results can be found in Table 7. Ultimately, on the SODA10M dataset, there was a 3.93% increase in AP50 and a 4% increase in mAP compared to the baseline. On the CBIA8K dataset, there was a 2.24% increase in AP50 and a 5.39% increase in mAP. Simultaneously, the increase in parameters and GFLOPs remained within acceptable ranges. Although the model detection speed has dropped slightly, it is still greater than 100 frames per second, which meets the real-time requirements. The model ultimately achieves the perfect balance of accuracy and speed.

**Table 7:** Detailed ablation experiment results on the SODA10M dataset and CBIA8K dataset.

Dataset	MSCM	BCAM	LD-Head	Dy-Loss	Adapt-ratio	AP50	mAP	Params	GFLOPs	FPS
SODA10M	–	–	–	–	–	67.98	42.38	7.02	15.8	151
	✓	–	–	–	–	69.14	43.13	6.92	15.5	139
	✓	✓	–	–	–	69.50	43.12	6.99	15.7	135
	✓	✓	✓	–	–	70.89	45.36	8.55	29.2	105
	✓	✓	✓	✓	–	71.10	45.78	8.55	29.2	104
	✓	✓	✓	✓	✓	71.91	46.38	8.55	29.2	104
CBIA8K	–	–	–	–	–	92.06	67.44	7.01	15.8	165
	✓	–	–	–	–	92.96	68.20	6.91	15.5	157
	✓	✓	–	–	–	93.58	68.23	6.98	15.7	143
	✓	✓	✓	–	–	93.71	68.52	8.55	29.2	117
	✓	✓	✓	✓	–	93.24	70.06	8.55	29.2	119
	✓	✓	✓	✓	✓	94.30	72.83	8.55	29.2	115

Compared with the baseline, with the continuous addition of improved modules, the detection accuracy of the detector has steadily improved. At the same time, it meets the real-time requirements. The above experiments show that the design of all our modules and structures is reasonable and practical. Some visual comparison results are shown in Figure 1.

## 5 Conclusion and outlook

In this paper, we provide a detailed exposition of the proposed ESF-YOLO, elucidating the relevance and effectiveness of the various designs through experimental validation. Specifically, a novel MSCM is introduced as the fundamental module of the backbone, enhancing the capability of feature extraction and fusion in the neural network. Furthermore, a new attention mechanism, BCAM, is incorporated to make the network more attentive to the detailed features of objects. This enables the network to distinguish different objects by leveraging differences in detail information, thereby enhancing its resistance to occlusion. Additionally, a lightweight Decoupled Head, LD-Head, is designed to significantly reduce the computational load of the neural network model. Finally, a redesigned loss function is proposed to address issues present in the original loss function, along with the introduction of an adaptive IoU ratio. ESF-YOLO demonstrates comprehensive superiority over the baseline model across various performance metrics, showcasing robust performance. In the future, we will continue to explore in the hope of implementing and optimizing the proposed ESF-YOLO on performance-constrained edge devices.

## Data availability statement

Publicly available datasets were analyzed in this study. This data can be found at: https://soda-2d.github.io/.

## Author contributions

WT: Writing – original draft, Writing – review & editing. XW: Writing – original draft, Writing – review & editing. TY: Writing – original draft, Writing – review & editing. ZL: Writing – original draft, Writing – review & editing. SW: Writing – original draft, Writing – review & editing.
